# Evaluating anticancer properties of Withaferin A—a potent phytochemical

**DOI:** 10.3389/fphar.2022.975320

**Published:** 2022-10-19

**Authors:** Maushma Atteeq

**Affiliations:** School of Systems Biology, George Mason University, Manassas, VA, United States

**Keywords:** Withaferin A, *Withania somnifera*, withanolides, apoptosis, cancer, Ashwagandha, cancer treatment

## Abstract

Withaferin A is a C28 steroidal lactone derived from the plant *Withania somnifera*, commonly known as Ashwagandha. Withaferin A has received great attention for its anticancer properties noted in cancer cells of various origins. Extracts of *Withania somnifera* have been used in traditional Ayurvedic and Unani Indian medicine for their various pharmacological benefits. In recent years, *Withania somnifera* or Ashwagandha extract has become popularized as a health supplement marketed for its stress and anxiety reducing effects. Withaferin A is one of the most studied withanolides extracted from *Withania somnifera* that has gained great attention for its anticancer, anti-inflammatory, metabolic, and pro-apoptotic effects. Extensive *in vivo* and *in vitro* studies have depicted Withaferin A’s interactions with key role players in cancerous activity of the cell to exert its pro-apoptotic effects. Withaferin A interactions with NF-κB, STAT, Hsp90, ER-α, p53, and TGF-β have noted inhibition in cancer cell proliferation and cell cycle arrest in G2/M stage, ultimately leading to apoptosis or cell death. This review highlights pro-apoptotic properties of Withaferin A including generation of reactive oxidative species, Par-4 activation, endoplasmic reticulum stress (ER) induction, and p53 activation. Analysis of Withaferin A’s involvement in various oncogenic pathways leading to malignant neoplasm and its pharmacologic activity in conjunction with various cancer drugs provides promising evidence in therapeutic potential of Withaferin A as a cancer treatment.

## 1 Introduction

Advances in science and medicine have dramatically impacted cancer treatments, increasing life expectancy of many individuals facing grim diagnosis of cancer. Despite medical advances, the disease still affects many individuals globally and is among the leading causes of death. Furthermore, drug resistance poses a serious problem that affects cancer cell survival and relapse. Therefore, development of potent therapeutics capable of impacting tumor progression and cancer cell proliferation is of crucial importance. Withaferin A, a phytochemical derived from plant *Withania somnifera* has been receiving great attention due to its anticancer properties observed in various mice models and tumor cell studies. Research on the compound depicts strong potential in development of Withaferin A as a therapeutic for cancer treatment.

Extracts from various parts of *Withania somnifera* plant have been studied for therapeutic effects in cancers of various origin. The anticancer effects of the plant were determined in the study by Khazal et al. (2014), which noted a reduction in mammary carcinomas in mice treated with root extract of *Withania somnifera* (Khazal et al., 2014). In another study, root extracts of *Withania somnifera* was used to treat human umbilical vein endothelial cells (HUVEC) and Withaferin A was identified as the active compound which inhibited cell proliferation (Mohan et al., 2004). Extracts from *Withania somnifera* leaves have also been used as treatment in studies investigating its anti-cancerous properties. In hepatocellular carcinoma cell line, HepG2 cells treated with leaf extract were found arrested in G0/G1 and G2/M phases with 61.9% in the former and 1.3% in latter phase; whereas the untreated cells were found in 2n phase (Ahmed et al., 2018). Cell cycle arrest was also noted in microglial cells treated with leaf water extract of *Withania somnifera* (Gupta and Kaur, 2016).

Withaferin A is a C28 steroidal lactone derived from the plant *Withania somnifera* of family Solanaceae; the plant is also known as Ashwagandha or Indian winter cherry. *Withania somnifera* is commonly used in ayurvedic medicine for enhancing neurological function, improving memory, reducing stress, and for promoting reproductive balance (Singh et al., 2021). Withaferin A is one of the major withanolides present in *Withania somnifera*. The withanolide component of Ashwagandha, Withaferin A, has been noted to possess antidiabetic, anti-epileptic, anti-inflammatory, anti-depressant, anti-arthritic, and anti-cancer properties (Behl et al., 2020). Withanolides have an ergostane back bone in their structure comprising the lactone ring at C-8 or C-9 side chain (Dutta et al., 2019). The extract of *Withania somnifera* or Ashwagandha is marketed as an herbal supplement for benefits of the compound as an adaptogen. The anti-cancer activity of Withaferin A has been widely studied in cancers of various origin and has been found to be a potent anti-carcinogen for multiple cancers such as, breast, lung, colon, brain, cervical, etc. (Dutta et al., 2019). Withaferin A has been widely studied *in vitro* and *in vivo* for its therapeutic potentials against cancer cells. Various studies investigating Withaferin A’s effects on oncogenic pathways have shown effectiveness against tumor cells and have determined the compound as a potential therapeutic for cancer.

## 2 Withaferin A characteristics

Structural modifications of Withaferin A play an important role. Chemical modifications such as, hydroxylation or acetylation can enhance the pharmacological activity of Withaferin A (Behl et al., 2020). The steroidal structure of Withaferin A contributes to its antiangiogenic properties (Rasool et al., 2017). The ring modified derivatives of Withaferin A can be studied for its anticancer properties; one such derivative is 3-azido-analogue which was successfully developed and exhibited strong anticancer properties (Rasool et al., 2017). Methylation of Withaferin A significantly influences its protein binding efficacy and results in chemotherapeutic attenuation (Behl et al., 2020). Withaferin A interferes with the interaction between p53 and Mortalin, a chaperone that can deactivate the tumor suppressor p53, induce deregulation of apoptosis, and promote carcinogenesis (Huang et al., 2015). Bioinformatics and experimental studies done by Huang et al. (2015) demonstrated that 3β-methoxy Withaferin A derivative to have a weaker binding interaction with its molecular targets, attenuating anticancer activity, compared to the non-modified form (Huang et al., 2015). This signifies importance of the appropriate structure of Withaferin A necessary for biologically important activities.

Withaferin A binding potential and interaction to the catalytic site of BCR-ABL, an oncogenic protein promoting cell proliferation and inhibition of apoptosis in chronic myeloid leukemia (CML), was determined as a result of computational analysis (Malik et al., 2022). Withaferin A interactions with ABL were found at the protein’s allosteric and catalytic site; furthermore, higher binding energy was determined for Withaferin A when compared to Imatinib and Ascinimib which are the clinically used drugs (Malik et al., 2022).

### 2.1 Bioavailability

Bioavailability and pharmacokinetic analysis of Withaferin A are important factors to account for when considering use of the compound as a therapeutic. The pharmacokinetic analysis profiles the targeted or untargeted metabolites post oral administration of one chemical component of the crude compound (Behl et al., 2020). One such analysis was done on mice plasma post oral administration of 1000 mg/kg of aqueous root extract of plant *Withania somnifera* showing that 0.4585 mg/kg of Withaferin A was present (Behl et al., 2020). This is suggestive of low bioavailability of Withaferin A when administered orally from aqueous solution of the crude extract. The bioavailability evaluation of withanolide constituents in *Withania somnifera* was performed *via* an *in vitro* absorption model system using canine kidney cell culture (Devkar et al., 2015). When compared to other withanolides present in *Withania somnifera,* Withaferin A was found to be the least permeable despite it being biologically active (Devkar et al., 2015). Non-polar withanolides with low molecular weights such as, withanolide A, withanone, withanolide B, and 1,2-deoxywithastramonolide were found to be the most permeable (Devkar et al., 2015). In another study, oral administration of aqueous *Withania somnifera* root extract in mice revealed 1.5 times greater relative bioavailability of Withaferin A compared to withanolide A (Patil et al., 2013).

A pharmacokinetic study was conducted by Modi et al. (2022), by orally administering 500 mg/kg dose of *Withania somnifera* root extract to male *Sprague Dawley* rats at 500 mg/kg and determined the absorption patterns of various withanolides, including Withaferin A (Modi et al., 2022). Rapid absorption of withanolide and withanoside constituents from the stomach was indicated by the results (Modi et al., 2022). The same study utilized an *in vivo* pharmacokinetics study which revealed a peak plasma concentration of Withaferin A of 124.415 ± 64.932 ng/ml over a maximum observed time of 0.250 ± 0.000 h; additionally, a time dependent absorption through the intestinal lumen depicting high solubility and permeability was observed (Modi et al., 2022). Among the seven constituents evaluated, only three, Withaferin A, Withanoside IV, and 1,2-deoxywithanstramonolide, were found to have increased oral absorption from the stomach lining, lower half-life, and lower oral clearance (Modi et al., 2022). Furthermore, Withaferin A and 1,2-deoxywithastramonolide constituents were detectable in plasma up till 10 h which depicted possibility of lower elimination rate for these constituents (Modi et al., 2022).

A study prepared the compound as a PEGylated nano liposomal Withaferin A (LWA) found improved bioavailability with this formulation of Withaferin A (Abeesh et al., 2021). The *in vitro* results from drug-release study demonstrated enhanced sustained drug release effect with LWA than with free Withaferin A (Abeesh et al., 2021). Another study by Dai et al. (2019) assessed oral bioavailability in male rats by administering Withaferin A as 5 mg/kg intravenously and 10 mg/kg orally (Dai et al., 2019). Results for oral bioavailability were 32.4 ± 4.8%, depicting low systemic absorption of the compound (Dai et al., 2019). The same study also conducted an *in vitro* analysis which revealed rapid depletion with half-life of 5.6 min of Withaferin A in liver microsomes; furthermore, the study verified first-pass metabolism by revealing 21% remaining Withaferin A over 1 h time period (Dai et al., 2019).

The bioavailability evaluation of withanolide constituents in *Withania somnifera* in an *in vitro* absorption model system using canine kidney cell culture was performed (Devkar et al., 2015). When compared to other withanolides present in *Withania somnifera,* Withaferin A was found to be the least permeable despite it being biologically active (Devkar et al., 2015). Non-polar withanolides with low molecular weights such as, withanolide A, withanone, withanolide B, and 1,2 deoxywithastramonolide were found to be the most permeable (Devkar et al., 2015).

### 2.2 Bioactivity and molecular interactions

**FIGURE 1:**
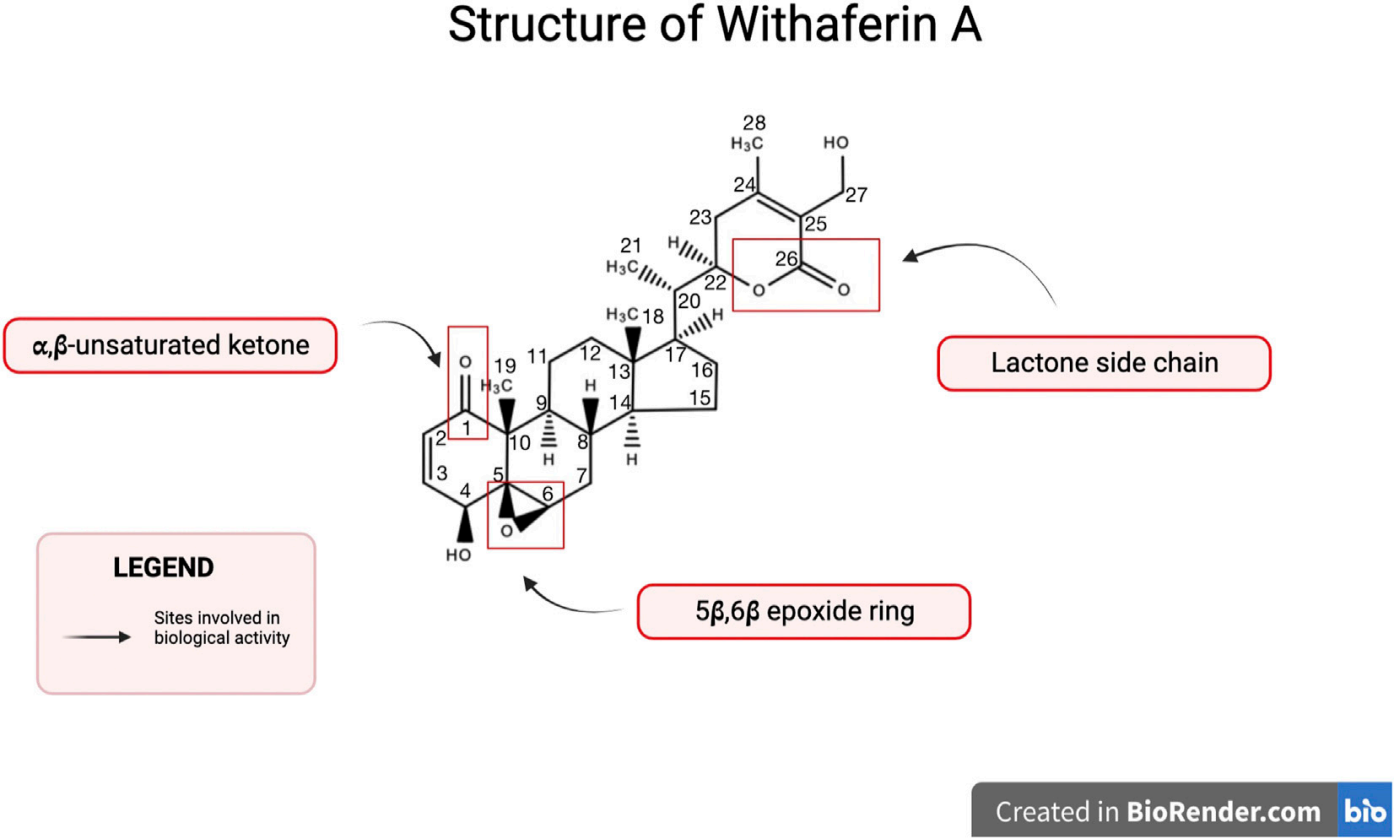
Structure of Withaferin A. Withaferin A is a C28 steroidal lactone with substituents important for biological activity. Notable substituents include a 5β,6β epoxide ring, a lactone side chain, and an α,β-unsaturated ketone.

Withaferin A is one of the major withanolides extracted from *Withania somnifera* and has been widely studied for its biologically active properties. Withanolides are characterized as a group of naturally occurring steroids with an ergostane skeleton containing C-28 (Verma et al., 2021). Specific carbons on the ergostane skeleton are oxidized, such as, C-26 and C-22 or C-26 and C-23, in order to form lactones or lactols that further classify the molecule as either Type A withanolide or Type B withanolide (Verma et al., 2021). The δ-lactones or δ-lactols constitute type A withanolides whereas γ-lactones or γ-lactols are classified as type B withanolide (Verma et al., 2021). The structure of Withaferin A along with these substituents is shown in Figure 1. Withaferin A is a type A withanolide such as, a steroidal lactone and it’s ring is subject to various oxidation patterns (Verma et al., 2021). Biological activity of Withaferin A is attributed to its key structural features, such as, a lactone side chain, α,β-unsaturated ketone, and a 5β,6β epoxide ring (White et al., 2016).

Withaferin A binding to Myc-Max-DNA and Mad-Max-DNA complexes has been observed where the binding analysis showed that bHLHZ domains of Myc and Mad proteins possess a possible ligand-binding site for Withaferin A (Yu et al., 2020). Withaferin A binding confirmation showed no interference with DNA nucleotides despite binding at the DNA-binding region of Myc and Mad; furthermore, a simulation analysis revealed that Withaferin A was highly stable with Mad and stabilized Mad-Max complex more than Myc-Max complex (Yu et al., 2020).

Recent study on structure-activity relationship and molecular dynamics of Withaferin A has revealed important findings regarding the phytocompound’s activity. The binding interactions of Withaferin A with the SARS-CoV2 main protease Mpro was investigated, and strong interactions were noted with polar and non-polar amino acid residues; notably GLN, THR, PRO, LEU, ALA, ARG, HIS, MET, GLY, and CYS (Ghosh et al., 2021). The study concluded Withaferin A as a strong potential inhibitor of SARS-CoV2 protease (Ghosh et al., 2021). Although unrelated to specific role players in cancer progression, these findings provide an important insight into the biologically active positions of the compound.

### 2.3 Toxicity

Safety assessment of Withaferin A and *Withania somnifera*, such as, testing animal toxicity, allows to determine whether the drug or compound use is safe in humans. This also provides information regarding the safe dosage of Withaferin A that can be administered for the desired effect. Animal toxicity studies have been conducted to assess safe use of Withaferin A. One such study assessed sub-acute toxicity of hydroalcoholic extract of *Withania somnifera* in Wistar rats concluded the compound as non-toxic (Prabu et al., 2013). Another study examined the acute and sub-acute toxicity of Withaferin A by oral administration of the *Withania somnifera* extract (WSE) in Wistar rats (Patel et al., 2016). For the acute toxicity study, a 2000 mg/kg dose was administered, for the sub-acute toxicity study, the Wistar rats were separated into groups by the quantity of oral administration of the *Withania somnifera* extract (WSE) which were, control, 500, 1000, and 2000 mg/kg of body weight/day for a period of 28 days (Patel et al., 2016). The study found no observed differences in body weight, organ weight, and found no histopathological differences due to treatment; furthermore, the serum chemistry revealed no toxicologically relevant differences that were statistically significant (Patel et al., 2016). Another study done in rats evaluated sub-toxicity of *Withania somnifera* used in combination with Ginseng reported emphysema and focal calcification in seminiferous tubules (Aphale et al., 1998). The study by Raut et al. (2012) evaluated the safety and activity of *Withania somnifera* in healthy volunteers of age 18–30 years by daily administration of *Withania somnifera* capsules with dosage increases every 10 days. The study found reduction in LDL and overall cholesterol levels, increased strength of muscle activity, and normal organ function levels (Raut et al., 2012).

Modi et al. (2022) performed an ADMET study on *Withania somnifera* extracts and assessed genotoxicity and mutagenicity at 500 g/kg dose. The ADMET study revealed none of the seven constituents of *Withania somnifera* extract, including Withaferin A, to be hepatotoxic, mutagenic, or carcinogenic (Modi et al., 2022).

Although Withaferin A was found to exert anti-proliferative effects against endometrial cancer KLE cells, a reduction in normal THESCs cells post 48 h prolonged treatment was observed with a decrease in viability from 100% to 25% (Xu et al., 2021). When compared, Withaferin A treatment showed greater toxicity towards endometrial cancer KLE cells than towards normal THESCs cells (Xu et al., 2021).

Another study screened Withaferin A for its cytotoxicity and antibacterial activity using various standard and multi drug resistant bacterial strains, and six tumor cell lines for cancers of various origin (Rossato Viana et al., 2022). These included lung cancer, glioblastoma, neuroblastoma, mouse melanoma, uterine colon cancer, and chronic myeloid leukemia (Rossato Viana et al., 2022). Withaferin A as found to have effective cytotoxic and antibacterial activity; cytotoxicity was observed *via* enhanced ROS production (Rossato Viana et al., 2022).

### 2.4 Effective dose and side effects

Information on the minimum effective dosage and potential side effects is crucial when considering use of Withaferin A as a therapeutic for cancer. In a phase I clinical trial conducted by Pires et al. (2020) safety and pharmacokinetics of Withaferin A was evaluated in patients with advanced stage high grade osteosarcoma; the study utilized oral administration of Withaferin A. The administered 400 mg capsule contained 18 mg of Withaferin A which was derived from the root extract of *Withania somnifera* (Pires et al., 2020). Adverse events noted in the study included fatigue, fever, rash, diarrhea, edema, and abnormal liver function tests (LFTs); a total of 11 adverse events were noted in 8 out of 13 enrolled patients in the study (Pires et al., 2020). Despite these side effects, the study found Withaferin A formulation to be well tolerated among patients (Pires et al., 2020).

In another study, Withaferin A treatment of human endometrial cancer KLE cells resulted in significant inhibition of cell proliferation and the IC_50_ of Withaferin A against these cells was noted to be 10 μM (Xu et al., 2021). Time-dependent and dose dependent anticancer activity of Withaferin A against KLE cells was observed depicting potent antiproliferative activity against the human endometrial cancer cells (Xu et al., 2021). Invasive cell percentage of KLE cells decreased from 100% at 0μM to 40% at 10 μM Withaferin A treatment. Oral administration of Withaferin A was found to reduce mammary tumor size in mice at dose of 1, 2, 3, 4, 8 mg/body weight three times per week (Sinha and Ostrand-Rosenberg, 2013).

### 2.5 Immunomodulatory effects

Among many properties of Withaferin A, its immunomodulatory effects are also of interest. Immunosuppressive effects of Withaferin A were noted in a study investigating long-term administration of compound as a treatment post clinical allogenic islet transplantation (Kumano et al., 2021). The study found significant reduction in mouse and human T-cell proliferation in a dose dependent manner and suppression of dendritic cell maturation with Withaferin A treatment; noting compound’s immunomodulatory effects (Kumano et al., 2021). In another study, 0.25–2.0 μg/ml Withaferin A treatment of mouse and human islets resulted in prevention of cytokine induced cell death and inhibited NF-κB activation (SoRelle et al., 2013). Immunomodulatory and anti-inflammatory effects of Withaferin A has been noted in macrophages and splenocytes (Alnuqaydan et al., 2022). Lipopolysaccharide (LPS) stimulated macrophages treated with Withaferin A showed reduced expression and secretion of the proinflammatory cytokines TNF-α, IL-1β, and IL-6 in a dose dependent manner (Alnuqaydan et al., 2022). Inhibitory immunomodulatory effects of Withaferin A were also noted in BALB/c mice spleen derived CD4/CD8 T cells and CD19 B cells in a dose dependent treatment (Alnuqaydan et al., 2022).

In a study on equines, Withaferin A was found to suppress equine neutrophil migration and suppress migration towards IL-8, LTB4, and PAF (Bayless et al., 2022). Immunomodulatory effects of Withaferin A are also observed in an *in vitro* study involving myeloid derived suppressor cells (MDSC) and tumor associated macrophages (TAMs) (Sinha and Ostrand-Rosenberg, 2013).

## 3 Anticancer properties

Withaferin A has been widely studied for its anti-cancer effects and multiple interactions contributing to its anti-cancer activity have been investigated. Many *in vivo* studies relating Withaferin A have demonstrated that the compound suppressed growth of the cancer cells derived from human tumors and of the experimentally cancer induced rodent models (Lee and Choi, 2016). Withaferin A has demonstrated ability to alter some of the major oncogenic processes such as, cell growth, migration, invasion, apoptosis, and neoangiogenesis, *in vivo* and *in vitro* (Vyas and Singh, 2014). Furthermore, Withaferin A has been noted to induce cell cycle arrest in cancer cells of various origin. Withaferin A treated breast cancer cells were found arrested in mitosis, this was noted by accumulation of securin and increased Ser10 phosphorylated histone H3 (Stan et al., 2008). Furthermore, Withaferin A treatment has also indicated G2-M cell cycle arrest in Caski cervical cell line as noted by dose dependent accumulation of the cyclin B1 which indicated cell cycle arrest in M phase (Mungala et al., 2011). Withaferin A treatment of human cervical cancer cells inhibited cell proliferation, induced p53 accumulation, and downregulated expression of HPV oncoproteins (Mungala et al., 2011).

Effects of Withaferin A treatment was studied in prostate cancer (PCA) cells utilizing SILAC-based proteomic approach where protein expression at 4 h and 24 h post exposure to Withaferin A was analyzed (Kumar et al., 2021). Upregulated expression of proteins involved in stress response pathways with prolonged exposure to Withaferin A was noted (Kumar et al., 2021). The treatment also showed an increase in oxidative stress, decreased mRNA translation, and increase in stress granule (SG) protein G3BP1 (Kumar et al., 2021). Although an increase in SG formation was noted with Withaferin A treatment, knockdown of G3BP1 expression was found to increase Withaferin A efficacy and reduce PCA cell survival (Kumar et al., 2021).

Efficacy of Withaferin A has also been studied in cancer cells that are able to replicate by developing an Alternate mechanism of Lengthening of Telomeres (ALT) (Yu et al., 2020). Although telomerase inhibitors are often used as anticancer drugs, they are found ineffective against ALT cancers which comprise approximately 15% of all cancers (Yu et al., 2020). In the study, isogenic cancer cells with or without telomerase were treated with Withaferin A where the greater cytotoxicity to ALT cancer cells was observed with Withaferin A treatment (Yu et al., 2020). Findings of the study further revealed that Withaferin A treatment led to telomere dysfunction, upregulation of DNA damage response, G2/M arrest and apoptosis in ALT cancer cells (Yu et al., 2020). In another study, Withaferin A was found to suppress human breast cancer cell viability in two different cell types, estrogen responsive MCF-7 and estrogen independent MDA-MB-231, in a dose dependent manner (Stan et al., 2008).

Anticancer effects of Withaferin A and its ability to modulate TGF-β signaling were investigated in human endometrial cancer where the results found G2/M cell cycle arrest and apoptosis of human KLE endometrial cancer cells (Xu et al., 2021). Withaferin A was found to inhibit human endometrial cancer cell proliferation *via* modulation of TGF-β signaling and by inhibiting TGF-β dependent Smad2 phosphorylation (Xu et al., 2021).

Selective activity if Withaferin A which contributes to compounds anticancer activity is of great interest due to notable interactions of the compound with cellular components/proteins involved in cancer progression and cell survival. Selective killing of cancer cells was detected in the study utilizing 20:1 ratio of withanone to Withaferin A, normal cells exposed to this treatment remained unaffected (Gao et al., 2014). It was determined that a ratio of 5:1 or 3:1 of withanone to Withaferin A was cytotoxic to both cancerous and normal cells (Gao et al., 2014).

### 3.1 Apoptosis

**FIGURE 2:**
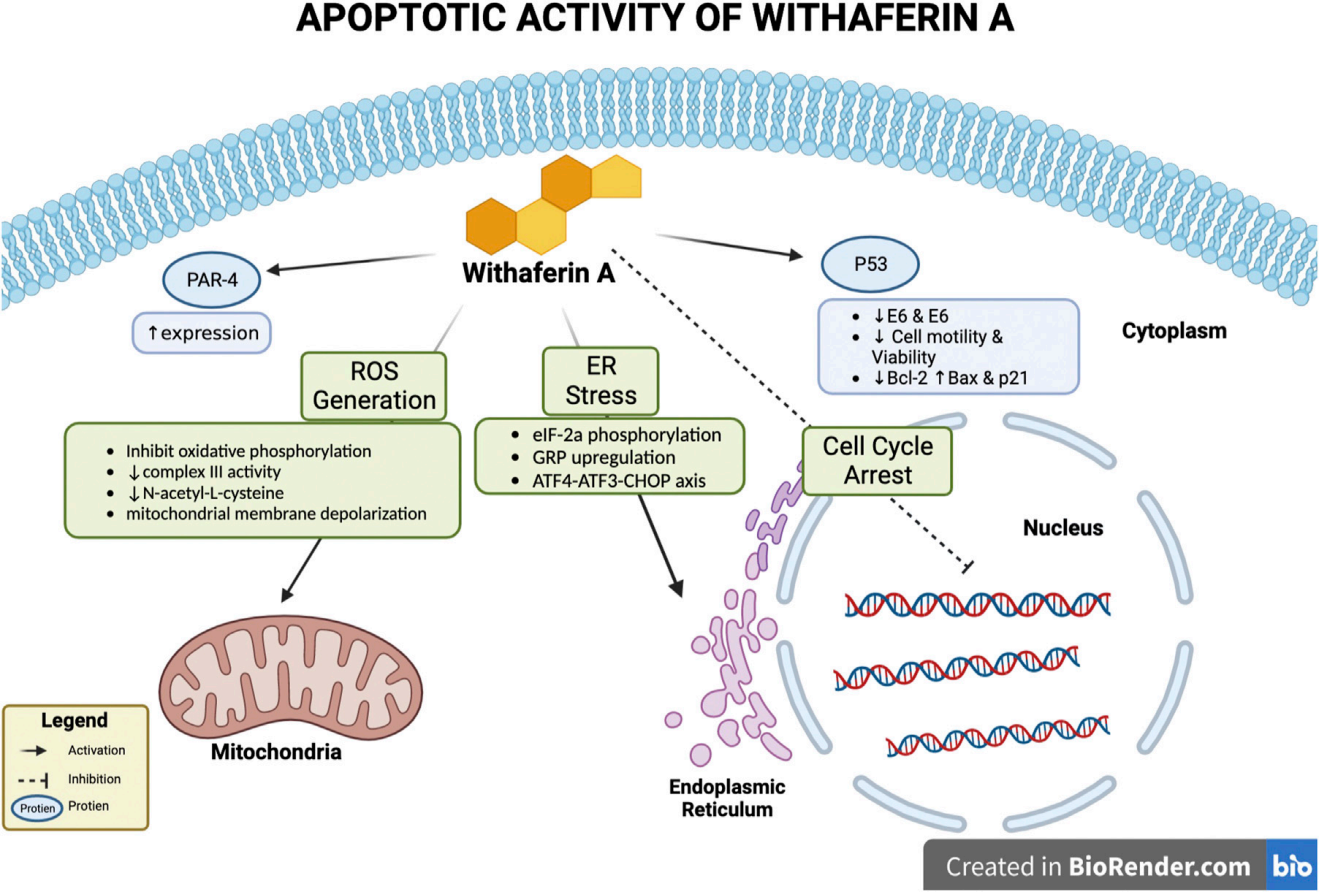
Apoptotic activity of Withaferin A. Withaferin A’s apoptotic activity can occur through various interactions with intercellular components/proteins. These include activation of Par-4 to increase its expression, activation of p53, endoplasmic reticulum (ER) stress induction, cell cycle arrest, and interaction with mitochondria to generate reactive oxidative species (ROS).

Withaferin A treatment has been noted to induce apoptosis *in vivo*, *in vitro*, or in both conditions for various cancers such as, breast cancer, cervical carcinoma, glioblastoma, melanoma, etc., (Vyas and Singh 2014). Various mechanisms by which Withaferin A induces apoptosis have been studied; these mechanisms depicted in Figure 2 include, involvement of reactive oxidative species (ROS), p53 activation, altering expression of pro and anti-apoptotic proteins, inducing endoplasmic reticulum stress (ER), activation of Par-4.

#### 3.1.1 Reactive oxidative species

A role of reactive oxidative species (ROS) is noted to contribute to Withaferin A induced apoptosis (Lee and Choi, 2016). This can be specific to the type of cells as Withaferin A treatment leads to ROS production by inhibition of mitochondrial respiration in human breast cancer cell line but not in normal human mammary epithelial cells (Hahm et al., 2011). In particular, the study noted that Withaferin A treatment inhibited oxidative phosphorylation and inhibited the activity of complex III (Hahm et al., 2011). Withaferin A treatment led to apoptosis in U266B1 and IM-9 human myeloma cell lines, inhibiting cell proliferation *via* ROS production (Li et al., 2022).

Generation of ROS was noted in prostate cancer cells treated with 3-azido Withaferin A (Rah et al., 2015). Furthermore, an increase in ROS production was observed with increasing concentration of 3-azido Withaferin A treatment and a decreased presence of the ROS inhibitor N-acetyl-L-cysteine (NAC) was noted (Rah et al., 2015).

Anticancer effects of low concentration of Withaferin A were investigated in oral cancer Ca9-22 cancer cells (Yu et al., 2020). Ca9-22 cells treated with Withaferin A at 0.5 µM over 24 h did not result in cytotoxicity, however, ROS generation, inhibition of wound healing, cell migration and invasion were observed (Yu et al., 2020). This study demonstrated that at low concentrations, Withaferin A induced ROS generation was below redox threshold depicting cellular survival and inhibition of cell migration; conversely, high Withaferin A concentration generated ROS at a higher level resulting in defined fate of cell such as, apoptosis (Yu et al., 2020). Hence Withaferin A induced ROS generation can lead to varying cellular response depending on the concentration administered. In another study, Withaferin A induced ROS leading to selective cell death was evaluated in oral cancer cells treated with varying concentrations of 1–3 µM (Chang et al., 2017). Higher ROS production in a dose dependent manner was noted in oral cancer cells (Ca9-22) treated with Withaferin A; additionally, G2/M cell cycle arrest, phosphorylated histone H2A.X based DNA damage, and mitochondrial membrane depolarization leading to cell death was also noted (Chang et al., 2017).

#### 3.1.2 p53 activation

Studies with Withaferin A have also shown to induce apoptosis by activating the tumor suppressor protein, p53, leading to cell cycle arrest and cell death in cancer cells (Lee and Choi, 2016). Results from several studies indicate an increase in p53 expression and phosphorylation of p53 at serine 315 residue with Withaferin A treatment (Lee and Choi, 2016). Withaferin A increased expression of p53 and p53 target genes p21 and Bax in human cervical cancer Caski cell line by decreasing the expression of human papillomavirus oncoproteins E6 and E7 which inhibit p53 (Mungala et al., 2011). In the same study, Withaferin A was found to suppress Bcl-2 in addition to inducing Bax; this led to PARP and caspase-3 cleavage and apoptosis in the Caski cells (Mungala et al., 2011).

Withaferin A treatment applied to different lung cancer cell lines revealed reduction in cell viability in a caspase-dependent manner and induced expression of p53 and Bax (Lin et al., 2021). The same study investigated the relationship between Withaferin A, p53, and two oncomiRs; miR-10b and miR-27a, the results indicated that Withaferin A decreases cell motility and viability by reducing miR-10b and miR27 (Lin et al., 2021).

#### 3.1.3 Endoplasmic reticulum stress induction

Withaferin A activity led to apoptosis in human renal carcinoma cell line (Caki) and in the androgen-insensitive prostate cancer cell lines by induction of endoplasmic reticulum (ER) stress. Withaferin A treatment of Caki cells led to induction of various ER stress markers which included phosphorylation of eukaryotic initiation factor 2a (eIF-2a), glucose-regulated protein (GRP) up regulation, and CAAT enhancer binding-protein homologous protein (CHOP) up regulation (Choi et al., 2011). Withaferin A is found to induce ER stress in glioblastoma cells *via* the ATF4-ATF3-CHOP axis and initiate apoptosis (Tang et al., 2019).

Induction of ER stress is noted in colorectal cancer (CRC) cells in a combination treatment of Withaferin A and 5-Fluorouracil resulting in autophagy and apoptosis was observed (Alnuqaydan et al., 2020). Furthermore, PCR analysis revealed an increased mRNA expression of BiP, CHOP, PERK, and eIF2α, which are ER stress markers, in CRC cells upon exposure to combination treatment of Withaferin A and 5-Fluorouracil (Alnuqaydan et al., 2020).

#### 3.1.4 Par-4 activation

Tumor suppressor protein, Par-4 selectively induces apoptosis in cancer cells in a p53 or PTEN independent manner, its constitutive expression is noted in normal human and rat cholangiocytes, however, in human cholangiocarcinoma, Par-4 expression declines (Lee and Choi, 2016). Furthermore, Par-4 expression is negatively correlated with cell proliferation markers and positively correlated with apoptotic markers in cholangiocarcinoma; additionally, Par-4 silencing *via* siRNAs lead to proliferation of cholangiocarcinoma cells in culture (Lee and Choi, 2016). Activation of Par-4 and its increased expression was noted in cholangiocarcinoma cells treated with Withaferin A (Lee and Choi, 2016).

### 3.2 Involvement in oncogenic pathways

**FIGURE 3:**
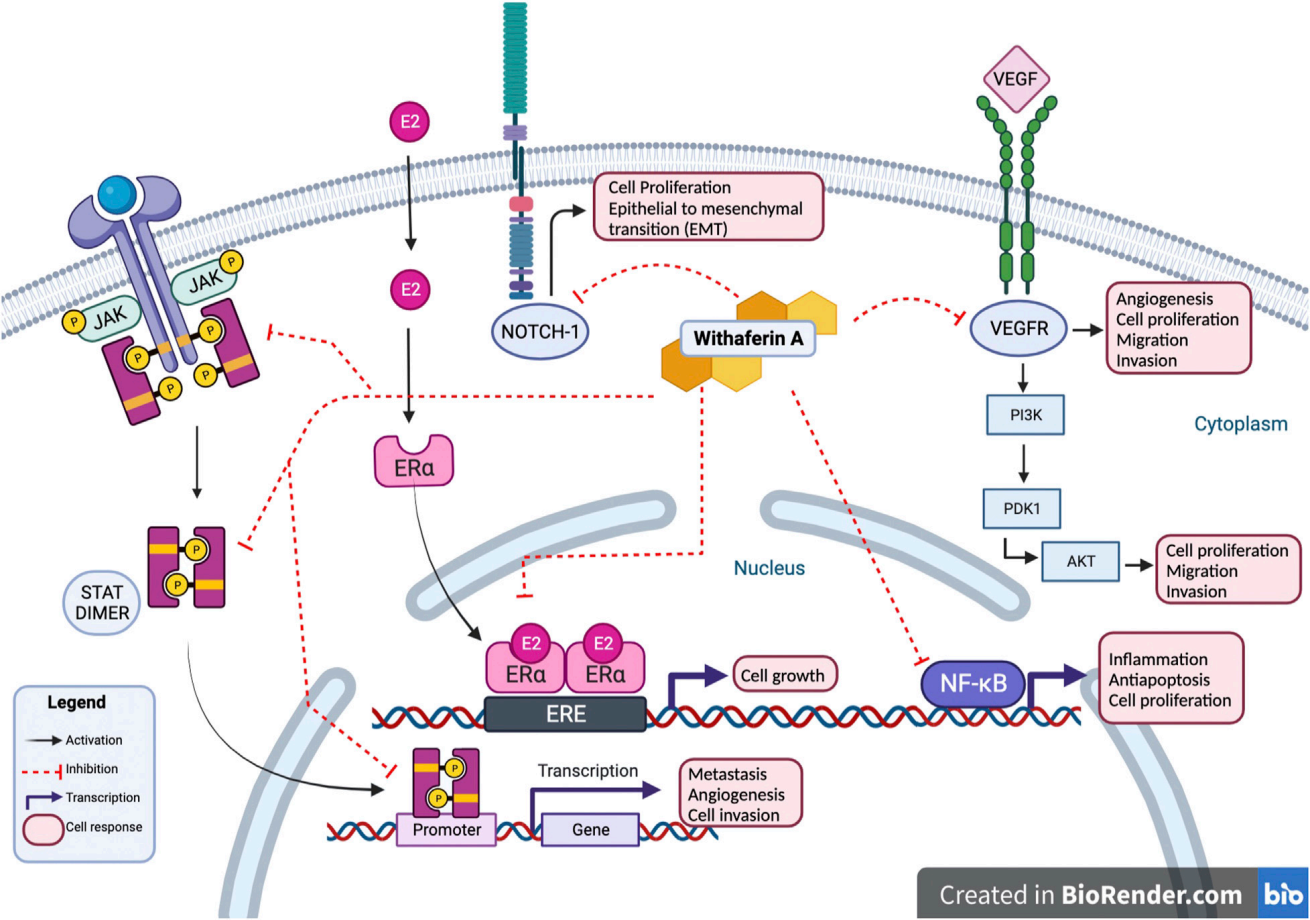
Withaferin A interactions. Withaferin A interactions with multiple intracellular proteins leads to modulation of cancerous activity of cell. Withaferin A has antagonistic activity with NOTCH-1, VEGFR, and JAK/STAT in cytoplasm. In cell’s nucleus, Withaferin A inhibits activity of transcription factors NF-κB, ER-α, and STAT3; preventing transcription of their downstream products. These interactions inhibit cancerous cell activity marked by cell proliferation, epithelial to mesenchymal transition, angiogenesis, cell proliferation, metastasis, and cell invasion.

Studies conducted on Withaferin A have noted the compound’s ability to target multiple oncogenic signaling pathways such as NF-κB, Akt, NOTCH, STAT3, and estrogen receptor α (ER-α); these pathways are often found active in human cancers (Vyas and Singh, 2014). Withaferin A interactions with JAK/STAT, NOTCH-1, VEGFR, ER-α, and NF-κB results in inhibition of cancerous activity of cell as shown in Figure 3. NF-κB is a transcription factor that is associated with inflammatory responses, Withaferin A treatment has been observed to inhibit NF-κB, suppress the nuclear translocation of its p65 subunit, and downregulate p65 in prostate cancer and soft tissue sarcoma cells (Vyasand Singh, 2014). Akt expression is associated with cell proliferation, migration and invasion, and epithelial to mesenchymal transition (EMT) in cancer cells; Withaferin A treatment suppressed Akt induced colorectal cancer (CRC) tumor growth in a xenograft model (Suman et al., 2016). Studies evaluating Withaferin A have noted the compound’s ability to modulate Notch signaling pathway in colon cancer and human breast cancer cells, ultimately impacting tumor’s progression (Lee and Choi, 2016). Withaferin A treatment is also noted to downregulate ER-α, a major therapeutic target in breast cancer treatment (Hahm et al., 2011).

Withaferin A treatment inhibited the growth of glioblastoma cells and induced apoptosis *via* up-regulation of Bim and Bad proteins (Tang et al., 2019). The same study revealed a novel pathway, the ATF4-ATF3-CHOP axis, which results in Withaferin A activation of apoptosis and cell cycle arrest at G2/M phase (Tang et al., 2019).

#### 3.2.1 JAK/STAT signaling

The JAK/STAT signaling pathway activation in various tumors has been found to contribute to cancerous properties of the cell and hence has been studied as a potential target pathway for developing cancer therapeutics (Thomas et al., 2015). Activation of STAT3 or STAT5 is typically associated with poor prognosis in cancers therefore development of effective cancer treatment focusing on suppression of STAT activity or inhibition of JAK/STAT pathway (Thomas et al., 2015). STAT3 is a major transcription factor associated with tumorigenesis due to aberrant regulation in cancer cells; it is found to promote cell invasion, metastasis, and angiogenesis *via* regulation of related genes (White et al., 2016). Withaferin A activity has been found to modulate the JAK/STAT pathway in human breast cancer cells by decreasing phosphorylation of STAT3 and JAK2 in MDA-MB-231 breast cancer cell line (Lee et al., 2010). The study noted inhibition of MDA-MB-231 and Interleukin-6 (IL-6) inducible activation of STAT3 and JAK2 with Withaferin A treatment (Lee et al., 2010). STAT3 levels were found decreased at 24 h time interval in Withaferin A treatment of 2 and 4 µM concentrations (Lee et al., 2010). Investigation on apoptotic effects of Withaferin A in human renal carcinoma Caki cells also note inhibition of STAT3 activation, inhibition of JAK2 phosphorylation, suppression of STAT3 regulated genes and its related anti-apoptotic proteins such as, survivin, Bcl-2, and Bcl-xl (Um et al., 2012). Withaferin A activity was found to decrease DNA binding activity of STAT3 in dose dependent manner by inducing STAT3 dephosphorylation (Um et al., 2012). Additionally, it was found that overexpression of STAT3 attenuated Withaferin A induced apoptosis of Caki cells (Um et al., 2012).

#### 3.2.2 NOTCH signaling

NOTCH signaling pathway has been widely studied as a potential target for developing cancer therapeutics. This is due to its role in controlling cellular proliferation, stem cell maintenance, apoptosis and modulating cell fate (Sail and Hadden, 2012). Aberrant NOTCH signaling leading to oncogenic functions has been noted in various solid tumors, such as, breast, colon, prostate, and pancreas, and is a key role player in affecting tumor progression *via* angiogenesis and promotion of epithelial to mesenchymal transition (EMT) (Raganathan et al., 2011). Effects of Withaferin A treatment in colon cancer cell lines have been observed where the bioactive compound has an inhibitory effect on NOTCH-1 signaling and downregulation of Akt/NF-κB/Bcl-2 pathways which promote cell survival (Koduru et al., 2010). Withaferin A treatment in human osteosarcoma cells was found to induce inhibition of cell growth and G2/M phase cell cycle arrest by inactivation of NOTCH-1 signaling and downregulating its downstream genes (Chen et al., 2014).

#### 3.2.3 Estrogen receptor α

The estrogen receptor α (ER-α) is heavily studied in cancers of various origin due to its role in cell proliferation and disease progression. ER-α plays a crucial role in driving breast and uterine cancers and is expressed in greater than 70% of ER + breast tumors (Porras et al., 2021). In MCF-7 human breast cancer cells, Withaferin A treatment resulted in reduction in ER-α protein levels and pS2 levels, a product of ER-α regulated gene; this was attenuated in E2 presence (Hahm et al., 2011b). A decrease in nuclear ER-α expression, anti-estrogen, and pro-apoptotic effects in response to Withaferin A treatment are observed in breast cancer cells (Hahm et al., 2011). In another study, Withaferin A treatment of MCF-7 breast cancer cells led to growth cell growth inhibition, cell cycle arrest in G2/M phase, and apoptosis associated with downregulation of ER-α, HSF1, RET and upregulation of phospho-p38 MAPK, p53, and p21 expression (Zhang et al., 2011).

#### 3.2.4 Nuclear factor kappa B

The transcription factor NF-κB plays a crucial role in controlling expression of regulatory genes for cell proliferation, cell death, and immunity (Zinatizadeh et al., 2020). Unregulated NF-κB activation leading to cancer cell proliferation is often observed in cancer cells; this suggests NF-κB signaling to be of interest for development of new therapeutics (Karin 2006). These functions of NF-κB drive great interest in its role in cancer cell activity and tumorigenesis. In a study evaluating effects of Withaferin A on rat C6 glioma cell line, anti-proliferative and proapoptotic effects were observed (Hou et al., 2017). The results depicted downregulation of Bcl2 protein expression and inhibition of Bax expression which point to inhibition of NF-κB transcription factor upon Withaferin A treatment (Hou et al., 2017). Withaferin A treated C6 glioma cells concomitantly suppressed TNF-α and inhibited nuclear translocation of NF-p65, leading to inhibition of NF-κB activity (Hou et al., 2017).

### 3.3 Inhibiting tumor angiogenesis and metastasis

Angiogenesis plays a crucial role in growth and metastasis of the malignant tumor and is often the targeted in therapeutic development for cancer treatment. Withaferin A has been noted to modulate angiogenic activity of the tumor *via* inhibiting activity of vascular endothelial growth factor (VEGF), a key regulator of angiogenesis. *In silico* molecular docking studies show favorable binding of Withaferin A with VEGF; results are comparable with Bevacizumab, an approved anti-cancer treatment (Saha et al., 2013).

In hepatocellular carcinoma cells, Withaferin A treatment inhibited cell proliferation, migration, invasion, anchorage independent growth and downregulated proteins associated with NF-κB, angiogenesis and inflammation (Shiragannavar et al., 2021). Furthermore, Withaferin A led activation of LXR-α results in inhibition of NF-κB and allows the phytochemical to modulate secretion of angiogenic factors and inflammatory cytokines (Shiragannavar et al., 2021).

Withanone and Withaferin A 20:1 combination treatment in HUVECs limited cell migration and invasion due to VEGF stimulation (Gao et al., 2014). Inhibition of cell migration and invasion was found to be comparable to Avastin treatment at 50 μg/ml depicting potency of anti-metastatic properties of the combination treatment (Gao et al., 2014).

Withaferin A led inhibition of tumor growth, metastasis, and angiogenesis was investigated in nude mouse model where Withaferin A was injected into the portal vein (Wang et al., 2015). Withaferin A treatment resulted in inhibition of liver tumor growth and decreased in incidence of lung metastasis (Wang et al., 2015). Treatment with Withaferin A also led to inhibition of Pyk2, ROCK1 protein, and VEGF expression (Wang et al., 2015). Anti-angiogenic property of Withaferin A was observed in Ehrlich ascites tumor cells where Withaferin A treatment led to decreased secretion of VEGF (Prasanna Kumar et al., 2009). Withaferin A is found to exert anti-angiogenic effects by inhibiting Sp1 transcription factor to VEGF-gene promoter, indicating the compound’s role in modulating tumor growth (Prasanna Kumar et al., 2009).

## 4 Drug interactions and potential as therapeutic

Various studies have noted great therapeutic potential of Withaferin A for treatment of various cancers. Multiple studies have also noted an increased sensitivity of cultured cancer cells in combination treatment with radiation and chemotherapy (Vyas and Singh, 2014). Treatment of human papillary and anaplastic thyroid cancer cells with Withaferin A in combination with Sorafenib resulted in synergistic effects *in vitro* and significantly induced apoptosis (Cohen et al., 2012). In another study on ovarian cancer, Withaferin A treatment in combination with low dose Cisplatin induced cell death and ROS generation, suggesting a potential for combination therapy (Kakar et al., 2012).

Synergistic effects of Withaferin A and 5-fluorouracil have been observed in a study involving colorectal cancer (CRC) cells where the combination treatment of the two substances leads to antiproliferation of the cells and induces endoplasmic reticulum (ER) stress, resulting in apoptosis and autophagy (Alnuqaydan et al., 2020). The antiproliferative potential of combination treatment was observed as the CRC cells were exposed to 0.1–100 µM of Withaferin A and 5-Fluorouracil resulting in reduction in cell viability in dose dependent manner (Alnuqaydan et al., 2020). The same study also revealed attenuation of expression of proteins associated with β-catenin pathway and cell cycle arrest in G2/M phase in colorectal cancer (CRC) cells (Alnuqaydan et al., 2020). Higher IC_50_ values of both compounds in normal colon cells was noted in comparison to CRC cells indicating a safe toxicity profile at a concentration where antiproliferative effects are observed (Alnuqaydan et al., 2020). The effects of combination treatment of Withaferin A and 5-flurouracil are of crucial importance as the currently available 5-flurouracil treatment is of concern due to its toxicity (Alnuqaydan et al., 2020).

A recent study investigated the potential of Withaferin A as a therapeutic for cancer treatment by incorporating the compound into nanosponges (NS) which serve to transport the compound to the target cell (Shah et al., 2021). SRB assay was utilized to evaluate the anticancer activity of Withaferin A-nanosponges (WA-NS) complex and the results demonstrated twice the efficacy against MCF-7 human breast cancer cells (Shah et al., 2021). WA-NS anticancer activity was compared to that of cisplatin and the results obtained were consistent as a 10 mg/kg WA-NS treatment at day 10 showed a reduction in tumor volume ∼72% which was consistent with ∼78% tumor volume reduction observed with cisplatin treatment of same dosage (Shah et al., 2021).

The *in vivo* tumor study utilizing LWA treatment, such as, PEGylated nanoliposome encapsulating Withaferin A, revealed reduction in tumor growth and improvement in survival for Dalton Lymphoma Ascites (DLA) tumor bearing mice; it was also revealed that LWA treatment reduced tumor growth by regulating expression of Ki-67 and cyclin D1 protein (Abeesh et al., 2021). Furthermore, the LWA formulation was found more effective when compared to non-encapsulated formulation as observed with a greater inhibitory effect in cancer cells *in vitro* (Abeesh et al., 2021). This depicts that the LWA formulation leads to a greater bioavailability of Withaferin A leading to enhanced drug effect than when administered as without nanoliposomal preparation (Abeesh et al., 2021).

Structural modifications to Withaferin A resulting in various derivatives have also been assessed for cytotoxicity against various cancer cells and demonstrate significant potential in development as a therapeutic for cancer. The 3-azido analogue of Withaferin A has exhibited 35-fold increase in cytotoxicity compared to its unmodified parent molecule (Yousuf et al., 2011). In prostate cancer cells, a low concentration of 3-azido Withaferin A (3-AWA) led to autophagy whereas administration of high concentration of 3-AWA resulted in the switch from autophagy to apoptosis (Rah et al., 2015).

## 5 Conclusion - evaluation of withaferin A as a potent anti-carcinogen

Withaferin A is being widely studied for its numerous benefits, a major one is its potential as a therapeutic for cancer treatment. Multiple *in vitro* and *in vivo* investigations have depicted promising outcomes for the compound’s anticancer effects when administered alone or in combination with other available therapeutics. Studies have shown anticancer effects such as, apoptosis, ROS generation, cell cycle arrest, etc. in cancers of various origins. Despite these results, an effective minimum dosage, toxicity, and bioavailability of the compound is yet to be thoroughly evaluated. Current studies depict low bioavailability of Withaferin A which poses a barrier in determining minimal dosage to have therapeutic effect on cancerous cells. However, efforts are also being made to overcome this barrier by studying various modes of administering Withaferin A and developing transporter methods that can lead to greater bioavailability of the compound such as use of nanosponges loaded with the biomolecule or development of PEGylated nanoliposomes. Additionally, delivery of Withaferin A using novel drug delivery approaches must be considered due to its low bioavailability. There is also a need to further evaluate adverse effects of Withaferin A in various populations such as immunocompromised, pregnant, or geriatric. Structural changes to Withaferin A and development of effective analogues that produce significantly potent effects may pose another opportunity for the molecule to be developed as a therapeutic agent. Although several studies have been conducted on Withaferin A and its interactions with various role players in oncogenic activity of the cell, further evaluation of Withaferin A’s activity must be made to develop the compound as an effective therapeutic.
